# Are there morphological and life‐history traits under climate‐dependent differential selection in S Tunesian *Diplotaxis harra* (Forssk.) Boiss. (Brassicaceae) populations?

**DOI:** 10.1002/ece3.3705

**Published:** 2017-12-15

**Authors:** Christoph Oberprieler, Claudia Zimmer, Manuela Bog

**Affiliations:** ^1^ Evolutionary and Systematic Botany Group Institute of Plant Sciences University of Regensburg Regensburg Germany; ^2^ Present address: General Botany and Plant Systematics Institute of Botany and Landscape Ecology Ernst Moritz Arndt University Greifswald Greifswald Germany

**Keywords:** adaptation, aridity, Cruciferae, phenotypic plasticity, population genomics

## Abstract

Adaptation of morphological, physiological, or life‐history traits of a plant species to heterogeneous habitats through the process of natural selection is a paramount process in evolutionary biology. We have used a population genomic approach to disentangle selection‐based and demography‐based variation in morphological and life‐history traits in the crucifer *Diplotaxis harra* (Forssk.) Boiss. (Brassicaceae) encountered in populations along aridity gradients in S Tunisia. We have genotyped 182 individuals from 12 populations of the species ranging from coastal to semidesert habitats using amplified fragment length polymorphism (AFLP) fingerprinting and assessed a range of morphological and life‐history traits from their progeny cultivated under common‐garden conditions. Application of three different statistical approaches for searching AFLP loci under selection allowed us to characterize candidate loci, for which their association with the traits assessed was tested for statistical significance and correlation with climate data. As a key result of this study, we find that only the shape of cauline leaves seems to be under differential selection along the aridity gradient in S Tunisian populations of *Diplotaxis harra,* while for all other traits studied neutral biogeographical and/or random factors could not be excluded as explanation for the variation observed. The counter‐intuitive finding that plants from populations with more arid habitats produce broader leaves under optimal conditions of cultivation than those from more mesic habitats is interpreted as being ascribable to selection for a higher plasticity in this trait under more unpredictable semidesert conditions compared to the more predictable ones in coastal habitats.

## 1 INTRODUCTION

The study of adaptation to abiotic and biotic factors of a species’ environment through the process of natural selection is paramount to our understanding of evolutionary change in morphological, physiological, and ecological traits. Besides the comparative approach to the study of adaptation based on macroevolutionary comparisons (e.g., Harvey & Pagel, 1991), microevolutionary studies of functional adaptation (adaptive evolution in functional traits within species) ultimately address the most proximal level of adaptation in functional traits because population‐level variation and its fitness consequences are assessed (Geber & Griffen, 2003). Local adaptation in functional traits through natural selection could be either studied by trait‐fitness correlations (selection differentials and selection gradients) in natural populations (Endler, 1986; Kingsolver et al., 2001), by field experiments such as common‐garden or reciprocal transplant experiments under assessment of trait and fitness, or by population genetic/genomic approaches (Savolainen, Lascoux, & Merilä, 2013). While in the two former method groups, the phenotypes are the starting point for selection studies and an assessment of fitness is necessary, the latter is less straightforward and starts with variation at the DNA level as a footprint of local adaptation, infers loci (so‐called outlier or candidate loci) that are under selection, and tests for statistically significant correlations of those loci with functional traits (Storz, 2005; Vasemägi & Primmer, 2005).

Studies on local adaptation are especially informative when clinal variation in functional traits is studied along environmental gradients, and alternative explanations for phenotypic clines (e.g., population demography, phylogeographical patterns) are excluded (Keller et al., 2009; Keller & Taylor, 2008; Savolainen et al., 2013). Among the different clinally varying environmental variables, aridity has been in the focus of several studies on local adaptation in the last years, mainly as a consequence of questions raised in conjunction with expected climatic changes and their impact on the vulnerability of plant species (e.g., Rehfeldt, Wykoff, & Ying, 2001). In a common‐garden experiment carried out in the Botanical Garden of Tel Aviv University (Israel), Petrů et al. (2006) found support for the expectation that desert populations have a much higher potential to respond plastically to differences in rainfall conditions. When irrigated, individuals of *Biscutella didyma* L. (Brassicaceae) from arid environments produced almost three times as many seeds as plants from the mesic Mediterranean. Therefore, while the former was considered following a strategy of maximizing reproduction during good years, a significantly higher investment of the latter populations into vegetative growth was interpreted as adaptation to aboveground competition in more stable and environmentally predictable habitats. A recent population genomic study of *Geropogon hybridus* (L.) Sch.Bip. (Compositae) along a steep precipitation gradient in C Israel revealed natural selection at 11 of 123 polymorphic loci studied that showed signs of putative precipitation‐related adaptation (Müller et al., 2017).

The coastal region of SE Tunisia exhibits steep gradients in precipitation, ranging from areas with mean annual precipitation values larger than 200 mm on the island of Djerba, over an approximately 20‐km broad strip parallel to the Mediterranean coast with precipitation values between 150 and 200 mm, to semidesert areas west of the more inland Matmata Mts with precipitation values of below 100 mm (Frankenberg & Klaus, 1987). Due to their elevation reaching more than 500 m a.s.l., the Matmata Mts themselves are again favored with mean annual precipitation values reaching those of lowland and coastal Djerba. As a consequence of these steep gradients, changes among vegetation types are pronounced and range between Mediterranean coastal vegetation (Djerba), over different steppe types in the coastal strip, to plant associations substituting *Juniperus phoenicea* woodland (Matmata Mts) and the semidesertic *Anthyllis sericea* steppe type at the eastern boundary of the Sahara desert (Frankenberg & Klaus, 1987). This constellation provides a suitable setting for studying climate‐dependent differential selection of morphological and life‐history traits along an aridity gradient.

The crucifer *Diplotaxis harra* (Forssk.) Boiss. (Brassicaceae) is a southern Mediterranean annual to short‐lived perennial herb with its distributional range also expanding to S Europe (Spain, Sicily) and SW Asia (Israel, Lebanon, Syria). Morphologically, it is easily characterized by its pendent siliquae with seeds in two rows. In North Africa, the species is widely distributed both in Mediterranean and in more arid, often sandy or gypseous areas with elevated salinity (Tlig, Gorai, & Neffati, 2008) and exhibits a broad range of ecological life‐cycle types with different reproductive strategies, ranging from ephemeral over modular monocarpic to coppiced polycarpic behaviors in dependence of the precipitation regime (Hegazy, 2001). In SE Tunisia, *Diplotaxis harra* is found along the above described, steep precipitation gradients and is therefore a suitable object for studying local adaptation in morphological and life‐history traits by means of a combined common‐garden and population genomics approach. Following the strategy of Herrera and Bazaga (2008), who pinpointed floral traits being under adaptive divergence in the Spanish hawk moth‐pollinated violet *Viola cazorlensis* Gand. (Violaceae), this study aims at the detection of amplified fragment length polymorphism (AFLP) loci under selection and asks whether these are correlated with phenotypic traits of interest, assuming that environment‐dependent selection in these morphological and life‐history traits has led to a selection signal in some of the AFLP loci via genetic hitchhiking.

## 2 MATERIAL AND METHODS

### 2.1 General strategy

Following the argumentation of Keller & Taylor (2008) and Keller et al. (2009), for the study of natural selection of phenotypic traits in wild populations, it is important to discriminate effects due to selection from changes caused by prior evolutionary history and chance events. Adopting the analytical sequence suggested by Herrera and Bazaga (2008) in their study of natural selection in flower characteristics of *Viola cazorlensis* Gand. (Violaceae), we first performed AFLP fingerprinting in populations of *Diplotaxis harra* in SE Tunisia in order to discriminate putative loci subject to selection from neutral ones. This was done with two methods (MCHEZA, Antao & Beaumont, 2011; BayeScan, Foll & Gaggiotti, 2008) that use locus‐wise measures of population differentiation (*F*_ST_ values) to find loci with differentiation values significantly larger than average as candidates for as being under divergent selection (“outlier loci”). Additionally, a third approach was used (as implemented in Samβada; Joost et al., 2007; Stucki et al., 2017) that searches for AFLP loci under selection by testing the strength of relationship between the presence or absence of an allele in a single‐individual genotype and environmental variables using logistic regression models. In a second step, we tested for geographical signals in the two nonoverlapping subsets of loci (“selected” vs. “neutral” ones) in order to detect isolation‐by‐distance (IBD) caused by demography and neutral history of populations (phylogeography) in the background of either natural selection along geographical gradients (“selected loci” with a significant IBD signal) or natural selection without a geographical motivation (“selected loci” without a significant IBD signal). Isolation‐by‐distance (IBD) was tested for using Mantel's test of correlation between a matrix containing geographical distances and a matrix with pairwise *F*_ST_ values among populations. Finally, we searched for correlations between life‐history and morphological traits and either of the two nonoverlapping subsets of loci (“selected” vs. “neutral” ones) by means of a Generalized Analysis of Molecular Variance (GAMOVA; Nievergelt, Libiger, & Schork, 2007). Here, we looked for traits being simultaneously correlated with the set of loci under selection and not correlated with the set of neutral loci because only in this constellation (scenarios 1.1 and 1.2 in Table 1) neutral variation as cause for phenotypic divergence among populations could be excluded and clinal or local selection could be assumed with confidence.

**Table 1:** Hypothetical outcomes from GAMOVA (Generalized Analysis of Molecular Variance; Nievergelt et al., 2007) tests addressing the correlation of quantitative traits with selected vs. neutral loci and the results of Mantel (Mantel, 1967) tests addressing geographical patterns in genetic differentiation of populations

Scenario	Test				Biological interpretation
	GAMOVA		Mantel test		
	Selected loci	Neutral loci	Selected loci	Neutral loci	
1.1	+	−	+	+/−	Phenotypic divergence of populations caused by selection along geographical cline
1.2			−	+/−	Phenotypic divergence of populations caused by local (nonclinal) selection
2	+	+	+/−	+/−	Phenotypic divergence of populations caused by neutral processes and (clinal or local) selectionNeutral variation cannot be excluded as cause for phenotypic divergence among populations
3.1	−	+	+/−	+	Phenotypic divergence of populations caused by neutral, geographical processes (IBD, founder effects, phylogeographical patterns)
3.2			+/−	−	Phenotypic divergence of populations caused by chance

+: denote significant, −: denote nonsignificant correlations.

### 2.2 Plant material and cultivation

The 12 populations of *Diplotaxis harra* surveyed in this study were sampled in Tunisia in March 2009 covering a climatic gradient between coastal Mediterranean conditions with relatively high precipitation and steppe vegetation in the east (Djerba) and semidesert areas with pronounced aridity in the west (Tozeur; see Table S1). In total, 182 individuals were sampled with 12–19 individuals per population. For each individual, leaf material was collected and dried with silica‐gel, and ripe fruits were stored in separate paper bags for the cultivation of half‐sib progeny in the Botanical Garden of Regensburg University.

Seeds from the 182 plant individuals sampled were sown on 5 March 2015 into a 1:1:2 mixture of sand, compost, and Einheitserde^®^ Classic (Sinntal‐Altengronau, Germany). After germination, five of the most vital half‐sib seedlings of each pot were selected and pricked out into larger pots with a 1:1 mixture of sand and Einheitserde^®^ Classic between 16 March and 25 March 2015 for further cultivation. On 8 April 2015, the five pricked‐out seedlings were reduced to a single one by removing the other four. While these first steps of cultivation were carried out in a glasshouse under a constant temperature regime (25°C), the further cultivation was carried out still under glass but under natural temperature conditions to avoid etiolation and rampant growth. During all steps of the experiment, we kept plants under identical light and water regimes, randomizing the pot positions every 3rd day. Due to germination failure in 16 seed samples from different populations, we propagated two or three half‐sib seedlings from 19 of single mother plants, respectively, in order to keep sample sizes of populations in balance. Therefore, in total, 187 seedlings were raised and scored for the life‐history traits and morphological characters described in the following.

### 2.3 Assessment of life‐history and morphological traits

Life‐history traits and morphological characters scored for each plant under cultivation are summarized in Table S2. In terms of life‐history traits, we recorded the number of days between sowing (March 25) and the opening of the first flower (variable *V01*) and the time between the starting of flowering and the formation of the first ripe fruits (*V03*). Plant architecture was described by scoring each plant for total height of the main shoot (*V09*) and the number of side shoots produced (*V10*). Additionally, the total number of leaves (*V07*)*,* the number of flowers along the main shoot (*V04*)*,* and the number of seeds per fruit (mean number of three siliques surveyed; *V06*) were assessed. For detailed morphometric analyses of leaves and flowers (described below), the sixth leaf produced along the main shoot was removed and dry‐pressed in a herbarium press, while a representative flower of each plant was removed and photographed keeping constant its dorsiventral orientation and adding a millimeter scale for calibration. For the assessment of leaf hairiness, a 5‐mm long stretch in the middle of each of the two lateral leaf margins was counted out for hairs, and the two values were averaged (*V08*).

### 2.4 Geometrical morphometrics of leaves and flowers

Size and shape of dried leaves were assessed after scanning with a scale for calibration. We used ImageJ v1.49 (Schneider, Rasband, & Eliceiri, 2012) for the measurement of the absolute area of the leaf lamina in cm^2^ (*V02*). For the comparison of leaf shapes, scanned‐in leaf silhouettes were imported into the outline analysis package *Momocs* (Bonhomme, Picq, Gaucherel, & Claude, 2014) in R v3.2.1 (R Development Core Team 2015), and an elliptical Fourier decomposition (Giardina & Kuhl, 1977; Kuhl & Giardina, 1982) was conducted. Following suggestions of Daegling and Jungers (2000), a preliminary analysis of all scanned leafs was carried out showing that the extraction of 15 harmonics was sufficient to describe 99% of the outline of even deeply dissected leaves. Subsequently, extracted Fourier harmonics were subjected to a principal component analysis (PCA) to gain PCA coordinates for each OTU as further three variables (*V15–V17*) for the statistical analyses.

In contrast to the Fourier approach for outline analysis of leaves, we followed a landmark/semilandmark approach for flower shapes as done in a comparable study in *Erysimum* (Brassicaceae) by Gómez, Perfectti, and Camacho (2006). Flower photographs from a standardized procedure (front view, planar position, retention of dorsiventral orientation) were overlain in CorelDraw^®^X8 (Corel Corporation, Ottawa, Canada) with four lines radiating from the center of the flower, each representing the midrib and length of a petal. Perpendicular lines at 25%, 50%, 75%, and 95% of the petal length and their points of intersection with the corresponding petal margin defined a total of five landmarks (Type I landmarks after Zelditch, Swiderski, Sheets, & Fink, 2004; flower center and petal tips) and 34 semilandmarks (Type II landmarks; petal outline semilandmarks). While flower size (*V05*) was represented in our analysis by the mean value for the four absolute petal lengths measured after calibration in ImageJ v1.49, flower and petal shape were analyzed by a Generalized Least Squares (GLS) Procrustes superimposition of landmarks/semilandmarks after their transfer from ImageJ v1.49 to the R package *geomorph* (Adams & Otarola‐Castillo, 2013), followed by a principal component analysis (PCA) for assessment of variation among OTUs and extraction of the main components responsible for shape differences. Again, PCA coordinates (first four axes) for each OTU were used as further morphological variables (*V11–V14*) in the statistical analyses.

### 2.5 DNA extraction, AFLP fingerprinting

Total genomic DNA was extracted using a CTAB (cetyltriammonium bromide) method. For extraction of total genomic DNA, c. 25 mg of leaf material was ground by mortar mill MM 301 (Retsch) and incubated for 15 min at 37°C and 15 min at 75°C in 700 μl CTAB buffer (with 2 μl β*‐*mercaptoethanol and 0.2 μl RNAse (100 mg/ml)). After cooling to room temperature and centrifugation for 2 min at 10,000 *g* at 4°C, 550 μl of the supernatant was combined with a mixture of phenol/chloroform/isoamyl alcohol (25:25:1; Rotiphenol, Roth), gently shaken for 2 min, and centrifuged for 5 min at 15,000 *g* and 4°C. 400 μl of the upper aqueous solution was carried over to a new vial and mixed for 1 min with 400 μl chloroform. After centrifugation (5 min, 15,000 *g*, 4°C), 400 μl of the upper aqueous solution was again transferred to a new vial and mixed well with 30 μl of a 3 M NaAc solution (pH 5). The DNA was precipitated by incubation for 30 min at 4°C and centrifugation for 20 min (20,000 *g*, 4°C). After removing the supernatant, the DNA pellet was washed with 800 μl cold ethanol (70%), dried in a speed‐vac, and finally dissolved in 50 μl Tris‐EDTA buffer.

Amplified fragment length polymorphism analyses generally adhered to the protocol described by Vos et al. (1995) and were carried out as described in Meister, Hubaishan, Kilian, and Oberprieler (2006), Meister, Kilian, and Oberprieler (2008) and Oberprieler, Meister, Schneider, and Kilian (2009) using *Mse*I and *Eco*RI restriction enzymes (MBI Fermentas), T4 DNA ligase (MBI Fermentas), and adaptors compatible with either of the two restriction sites. Restriction ligation was carried out at 37°C for 2 hr, after which the ligase was heat‐inactivated. Preselective amplification used primers with one selective nucleotide (C for the *Mse*I primer and A for the *Eco*RI primer), while the selective amplification used primers with further two selective nucleotides. After screening 64 different primer combinations in the selective PCR, the following three combinations were finally used: (D2) *Eco*RI‐AAC/*Mse*I‐CTA, (D3) *Eco*RI‐AAG/*Mse*I‐CTC, (D4) *Eco*RI‐ACA/*Mse*I‐CTA. All PCR reactions for the preselective amplification were carried out using the AFLP Amplification Core Mix (Applied Biosystems), while all reactions for the selective amplification were carried out with *Taq* polymerase (PeqLab). After a precipitation step, selective amplification products were separated via capillary gel electrophoresis using a CEQ 8000 automated sequencer (Beckman‐Coulter). Electropherograms were exported to GelCompar (Applied Maths Inc.). Using 24 duplicate DNAs and screening 168 combinations of parameters for the automated analysis of AFLP electropherograms according to Holland, Clarke, and Meudt (2008), we reached at an optimal parameter setting with “minimum profiling” set to 1.0 (D2) or 2.5 (D3, D4), “minimum area” set to 0.1 (D2) or 0.2 (D3, D4), and “matching tolerance” set to 0.2 (all three primer combinations). With these settings, the final analysis of all individual AFLP electropherograms was carried out in GelCompar (Applied Maths Inc.) producing a 0/1 matrix, upon which the downstream statistical analyses were based.

### 2.6 Analyses of population structure

We analyzed the binary matrix from AFLP fingerprinting by a principal coordinates analysis (PCoA) based on pairwise Bray–Curtis distances among the 181 operational taxonomic units (OTUs) using the program MVSP v3.1 (Kovach, 1999). We further used a Bayesian clustering approach as implemented in the program Structure v2.3.3 (Falush, Stephens, & Pritchard, 2007; Pritchard, 2010; Pritchard, Stephens, & Donnelly, 2000). The optimal number of genetic clusters (*k*) was calculated according to the method of Evanno, Regnaut, and Goudet (2005) using *k* values ranging from 1 to 9 with ten repetitions for each *k*. The data sets ran using the admixture model with 150,000 generations after 150,000 burn‐in generations.

### 2.7 Search for loci under selection

We have used three different approaches to search for AFLP markers that are either under direct selection themselves or closely linked to loci under selection. As the first two frequentist *F*_ST_ outlier detection methods (i.e., MCHEZA, BayeScan) make the assumption of populations being in Hardy–Weinberg equilibrium and we were unaware about the realization of this prerequisite in the populations under study, we applied a third spatial analysis method (Samβada) that does not use a population genetic approach.

1. The program MCHEZA (Antao & Beaumont, 2011) is based upon the Dfdist kernel which searches for loci under selection based on the principle that genetic differentiation among populations is expected to be higher for loci under divergent selection than for the rest of the genome (Beaumont & Balding, 2004; Beaumont & Nichols, 1996). The program calculates locus‐wise allele frequencies and *F*_ST_ values using the Bayesian method of Zhivotovsky (1999) and uses computer simulations to produce *F*_ST_ values for modeled AFLP loci under neutral conditions. As suggested by the software developers, we used the “neutral mean *F*_ST_” and the “force mean *F*_ST_” options to calculate a “trimmed mean *F*_ST_,” which is then used as an estimate of the average “neutral” *F*_ST_ uninfluenced by outlier loci. Loci with a noticeable high *F*_ST_ value were considered as being under divergent selection. The analysis was performed using 1,000,000 simulations, a 0.95 confidence interval (CI), and a false discovery rate (FDR) of 0.05 to correct for multiple testing. For all other parameters, we used the default values.
2. The program BayeScan v2.1 (Foll & Gaggiotti, 2008) was used to check for the reliability of the outlier loci found with MCHEZA owing to the fact that overall *F*_ST_ values calculated by MCHEZA may indicate exaggerated population differentiation caused by a single or a few populations with extreme allele frequencies. BayeScan uses a regression approach and a hierarchical Bayesian procedure to simultaneously estimate *F*_ST_ values for every locus in each population. For this analysis, we started with 50 pilot runs of 5,000 iterations each and finally ran a total number of 250,000 iterations with a burn‐in of 50,000 and a thinning interval of 20, resulting in a sample size of 10,000 iterations. All other parameters were set to default. As above, we used a FDR of 0.05 for outlier detection.
3. A third method to detect adaptive loci is the spatial analysis method as implemented in the program Samβada (Joost et al., 2007; Stucki et al., 2017). In contrast to the two above‐mentioned procedures, it does not analyze AFLP data in a population genetic framework but is based on logistic regression models testing the strength of relationships between the presence or absence of an allele/band in a single‐individual genotype and environmental variables. As explained by Joost et al. (2007), this procedure has some advantages over the population genetic approaches by not being dependent on any assumption of inbreeding coefficients, therefore being applicable to sampling designs with only single individuals per location, and immediately returning direct relationships between environmental variables and candidate loci under selection coupled with these variables. Two statistical tests [a log‐likelihood ratio test and the Wald test (Wald, 1943)] are implemented to evaluate the significance of the correlation between the presence/absence of an AFLP band at a locus and an environmental variable. We ran univariate models including the 20 climatic variables extracted for the 12 sampled populations (Table S3) and used a significance threshold of 0.05, which was Bonferroni corrected for multiple comparisons. Aiming at a reduction in complexity, we additionally submitted the mentioned 20 climatic variables (together with two further variables giving the latitude and longitude of sample localities) to a principal component analysis (PCA) and added the first three extracted principal components *(PC1‐PC3)* as further variables to the Samβada analyses.

### 2.8 Correlation of selected and neutral loci with geography

After assignment of loci to one of the two nonoverlapping subsets of loci (“selected” vs. “neutral” ones) according to the results of the MCHEZA, BayeScan, and Samβada analyses, respectively, we performed tests for isolation‐by‐distance (IBD) in each of the resulting locus subsets by Mantel tests (Mantel, 1967) based on matrices of pairwise population differentiation (Φ_PT_‐values from Analyses of Molecular Variance) and geographical distances among populations. Analyses of Molecular Variance (AMOVAs) and Mantel tests were carried out in GenAlEx v.6.5 (Peakall & Smouse, 2012).

### 2.9 Testing for selection on life‐history and morphological traits

A Generalized Analysis of Molecular Variance (GAMOVA; Nievergelt et al., 2007) was used to test for signature of selection on life‐history and morphological traits. GAMOVA was carried out with the R program package *vegan* v.2.3‐0 (Oksanen et al., 2015) and was based on pairwise Φ_PT_‐values among populations from Analyses of Molecular Variance (see above) and population‐wise arithmetic means of the 17 characters representing life‐history and phenotypic traits. By doing two independent GAMOVAs based on pairwise Φ_PT_‐values among populations carried out with the two nonoverlapping subsets of loci (“selected” vs. “neutral” ones), we were able to discriminate among life‐history and morphological traits that showed significant correlations with (1) selected, (2) neutral, (3) both, and (4) none of the two‐locus subsets. Statistical significance of the relationships was tested with permutation tests based on 9,999 repetitions.

## 3 RESULTS

### 3.1 Variation in life‐history and morphological traits

Boxplots of all life‐history characters (i.e., *V01*: time between sowing and opening of the first flower; *V03*: time between the starting of flowering and the formation of the first ripe fruits) and all nonshape morphological traits (*V02, V04–V10*) are depicted in Figure S1, which also contains information on significant differences among populations in these traits. No significant differences among populations were found for characters *V02* (absolute area of the lamina of the sixth leaf on a plant), *V04* (number of flowers along the main shoot), and *V06* (number of seeds per silique), while in all other characters measured differences between at least two populations reached significance.

**Figure 1:**
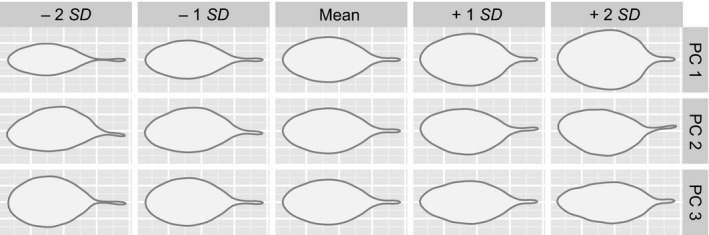
Results of a principal component analysis (PCA) based on the extraction of 15 Fourier harmonics describing the outline shape of the sixth cauline leaf. For each of the three extracted principal components (PC 1 to PC 3) explaining more than 5% of the total variance, the variation of leaf shape is depicted with five representative leaf silhouettes along the axis concerned. While PC 1 explains 54.7% of the total variance, PC 2 with 12.4% and PC 3 with 9.5% are considerably less important than the first axis

Geometric morphometrics of leaf shape based on a Fourier transformation with 15 harmonics followed by a principal component analysis (PCA) resulted in three principal components explaining more than 5% of the total variance. While principal component PC1 accounted for 54.7% of the total variance, the following two components showed considerable lower values for variances explained (12.4% and 9.5%, respectively). As demonstrated in Figure 1, PC1 is mainly influenced by the length‐width ratio of the leaf lamina, PC2 by the symmetry of leaves, and PC3 by the shape of the leaf apex (rounded vs. pointed), while higher harmonies describing leaf dissection contribute only to principal components with even lower importance (<5% of total variance). The 12 populations surveyed differed in their variability along the first two axes (Figure S2) but showed only little differences among them; the extremes being represented by populations 1 (Djerba) and 6 (NE Tataouine) on the one hand (broad leaves) and populations 11 (Tozeur) and 12 (Selja) on the other (narrow leaves).

**Figure 2:**
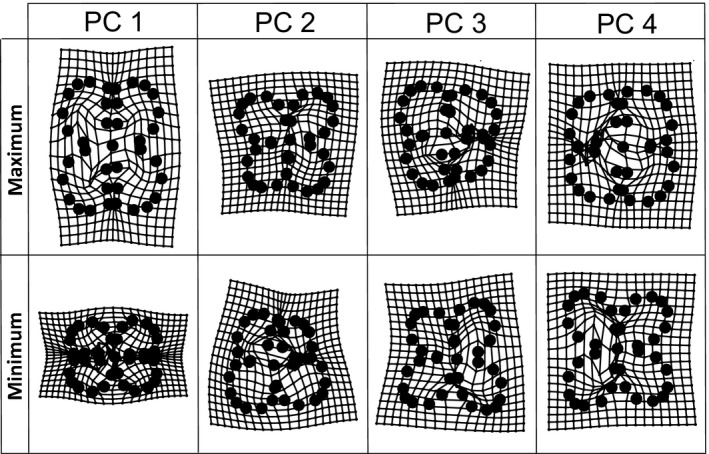
Results of a principal component analysis (PCA) based on Generalized Least Squares (GLS) Procrustes superimposed coordinates of 37 flower landmarks/semilandmarks. For each of the four extracted principal components (PC 1 to PC 4) explaining more than 5% of the total variance, the variation of flower shape is depicted with two thin‐plate spline flower reconstructions at the extremes of the axis concerned. PC 1 accounts for 36.7%, PC 2 for 17.1%, PC 3 for 16.4%, and PC 4 for 10.7% of the total variance

The Generalized Least Squares (GLS) Procrustes superimposed coordinates of flower landmarks/semilandmarks analyzed by a principal component analysis (PCA) resulted in four principal components, each one explaining a minimum of 5% of the total variance in the data set. As the thin‐plate spline flower reconstructions for the extreme values of the four principal components show (Figure 2), PC1 accounting for 36.7% of the total variance mainly describes the shape of the flower (broad vs. narrow along median axis), while PC2 (17.1%) represents differences in flower dorsiventrality, PC3 (16.4%) in flower asymmetry, and PC4 (10.7%) in the degree of petal overlap. The 12 populations surveyed showed considerable differences in the intrapopulation variability of flower shapes, but little differences among populations were observed (Figure S3); the extremes being represented by populations 3 (Dkhilet Toujane) and 8 (Ksar Hadada) on the one hand (narrow flowers) and populations 5 (Tamezret) and 7 (W Tataouine) on the other (broad, stout flowers).

### 3.2 AFLP fingerprinting and population structure

Amplified fragment length polymorphism fingerprinting of 181 individuals and 24 replicates with three selective primer pairs (E‐AAC/M‐CTA, E‐AAG/M‐CTC, E‐ACA/M‐CTA) and an optimized, automated band scoring (Holland et al., 2008) yielded 732 marker bands (260, 223, and 249, respectively) in the range between 100 and 420 bp length. An Euklidian error rate of 9% was estimated among all replicates, being a high but reasonable rate for an automated band scoring procedure compared to a manual one (Holland et al., 2008); 23 of the 24 replicates were correctly paired when analyzed together with the remaining OTUs.

**Figure 3:**
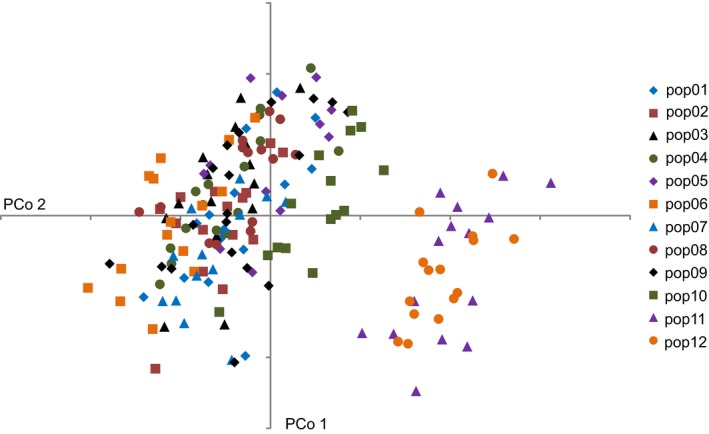
Ordination of OTUs in the two‐dimensional space of the first two axes of a principal coordinates analysis (PCoA) based on AFLP fingerprint data and pairwise Bray–Curtis distances among OTUs, with PCo 1 accounting for 6.1% and PCo 2 for 5.1% of the total variance. Signatures denote the 12 populations surveyed

A principal coordinates analysis (PCoA) based on pairwise Bray–Curtis distances among the OTUs resulted in the ordination depicted in Figure 3. While the first principal coordinate accounts only for 6.1% and the second for only 5.1% of the total variation, a clear subdivision of OTUs is observed, with members of populations 11 (Tozeur) and 12 (Selja) being removed from the rest of populations. The same bipartition of populations was found in the Structure analysis (Figure S4), in which *k *= 2 was found as the optimal cluster number, and population 10 (El Hamma) constitutes an intermediate link between the two groups of populations.

### 3.3 Analysis of bioclimatological data

The mentioned bipartition of sampled populations of this study found in the AFLP fingerprinting is mirrored in the climatological patterns. When bioclimatic variables (*bio01‐bio20*) for the 12 populations are subjected to a principal component analysis (PCA; Figure S5, Table S4), the first axis PC1 (accounting for 55.7% of the total variance in the data set vs. only 26.9% on PC2 and 11.5% on PC3) clearly separates populations 11 (Tozeur) and 12 (Selja) from the other populations, with population 1 (Djerba) forming the counterpart to populations 11 and 12 while the other localities are ordinated between population 1 and intermediate values along PC1. The high number of bioclimatic variables with high loadings on this axis indicates the marked differences in temperature and precipitation along the long gradient between Djerba (population 1) and the two populations west of Chott el Djerid (populations 11 and 12).

When these two outlier populations are removed from the analysis (Figure S6, Table S4), a more homogenous ordination of populations is observed together with a more balanced importance of extracted principal components (PC1: 48.1%; PC2: 36.7%; PC3: 9.6%). When the first two principal components are taken into consideration simultaneously, populations are found being arranged in a polarized manner between populations 1 (Djerba), 4 (Toujane), and 9 (Mareth) on the one side and 6 (Kasba Drina) and 7 (Tataouine) on the other one. Inspection of the loadings of bioclimatic variables on axes PC1 and PC2 indicates that these populations form the extremes in a gradient between relatively high precipitation, low temperature, and low seasonality/continentality at the former locations and relatively low precipitation, high temperature, and high seasonality/continentality at the latter ones.

### 3.4 Loci under selection

When all 12 populations under study were considered in the search for AFLP loci under selection, MCHEZA simulations resulted in a trimmed mean of *F*_ST_ = 0.0249 over all 732 loci (Table 2). Seventeen (2.3% of the total) fell outside the 95% and 14 (1.9% of the total) outside the 99.5% confidence interval constructed from the simulated distribution (Figure S7). While all of these (selected) loci showed *F*_ST_ values significantly greater than expected (range: 0.148–0.547; mean *F*_ST_ ± SE = 0.250 ± 0.027), the remaining 715 (neutral) loci exhibited smaller levels of differentiation (mean *F*_ST_ ± SE = 0.015 ± 0.0012). All except one (i.e., L514) of the outlier loci found with MCHEZA were corroborated as being outliers with the Bayesian regression method implemented in BayeScan, resulting in an intersection set of 16 method‐independent AFLP loci considered as being under directional selection or being linked to a locus under selection. The Samβada search for loci under selection resulted in 19 loci, for which there was a highly significant correlation detectable between the absence/presence of a band and a single or more environmental (bioclimatic) factors. Twelve of these loci were from the set of 16 outlier loci found with the above‐mentioned *F*_ST_‐based methods.

**Table 2:** Characterization of 26 outlier loci found as being under selection by one (italics)*,* two (simple), or three (bold) of the three methods implemented in MCHEZA, BayeScan, and Samβada, respectively, in the complete data set (pop01‐pop12). For each locus, estimates of *F*_ST_ and associated significance values (false discovery rate FDR) obtained using MCHEZA and BayeScan are given. Outlier loci found by Samβada are documented together with the environmental variable(s) showing significant correlations with the presence of the dominant allele

	Locus	MCHEZA		BayeScan		Samβada
		*F*_ST_	FDR	*F*_ST_	*q*‐value	
*D2*	*L025*	0.124	>0.05	**0.108**	**0.0037**	
**D2**	**L039**	**0.148**	**<0.05**	**0.081**	**0.0309**	*PC3*
**D2**	**L040**	**0.164**	**<0.005**	**0.119**	**0.0050**	*PC3*
**D2**	**L115**	**0.226**	**<0.005**	**0.108**	**0.0008**	*PC1, bio02, bio04, bio05, bio07, bio12, bio15, bio09, bio10, bio13, bio16, bio17, bio18, bio19*
**D2**	**L124**	**0.193**	**<0.005**	**0.108**	**0.0018**	*PC1, bio02, bio04, bio05, bio07, bio09, bio10, bio12, bio13, bio15, bio16, bio19*
D2	L139	**0.176**	**<0.005**	**0.089**	**0.0215**	
*D2*	*L141*	0.118	>0.05	0.032	0.3395	*bio15*
*D2*	*L145*	0.098	>0.05	0.048	0.1708	*bio15*
**D2**	**L184**	**0.547**	**<0.005**	**0.186**	**<0.0001**	*PC1, PC3, bio01, bio02, bio04, bio05, bio07, bio08, bio09, bio10, bio12, bio13, bio15, bio16, bio17, bio19*
**D2**	**L237**	**0.153**	**<0.05**	**0.140**	**0.0001**	*PC3*
D3	L288	0.150	>0.05	**0.131**	**<0.0001**	*PC1, bio15, bio17*
**D3**	**L302**	**0.395**	**<0.005**	**0.150**	**<0.0001**	*PC1, bio01, bio02, bio04, bio05, bio07, bio09, bio10, bio12, bio13, bio15, bio16, bio17, bio18, bio19*
**D3**	**L307**	**0.396**	**<0.005**	**0.154**	**<0.0001**	*PC1, bio02, bio04, bio05, bio07, bio08, bio09, bio10, bio13, bio15, bio16, bio17, bio19*
D3	L359	**0.169**	**<0.005**	**0.114**	**0.0024**	
D3	L365	**0.202**	**<0.005**	**0.124**	**0.0005**	
*D3*	*L452*	**0.155**	**<0.05**	0.067	0.0603	
**D3**	**L459**	**0.314**	**<0.005**	**0.257**	**<0.0001**	*PC1, bio02, bio04, bio05, bio07, bio12, bio13, bio16, bio18, bio19*
D3	L461	0.114	>0.05	**0.095**	**0.0164**	*bio10, bio12, bio13, bio16, bio19*
D3	L462	0.155	>0.05	**0.093**	**0.0111**	*PC1, bio05, bio09, bio10, bio19*
**D3**	**L466**	**0.247**	**<0.005**	**0.100**	**0.0069**	*PC1, bio01, bio02, bio05, bio07, bio09, bio10, bio12, bio13, bio15, bio16, bio18, bio19*
**D4**	**L487**	**0.270**	**<0.005**	**0.124**	**0.0012**	*PC1, bio02, bio05, bio09, bio10, bio13, bio15, bio16, bio19*
**D4**	**L511**	**0.279**	**<0.005**	**0.188**	**<0.0001**	*bio08, bio09, bio10, bio15*
D4	L514	0.147	>0.05	**0.159**	**<0.0001**	*bio15*
D4	L548	**0.217**	**<0.005**	**0.149**	**<0.0001**	
*D4*	*L617*	0.080	>0.05	**0.076**	**0.0409**	
*D4*	*L653*	0.085	>0.05	0.026	0.7468	*PC1, bio05, bio09, bio10*

When the search for loci under selection was based on the ten genetically more homogenous populations of SE Tunisia (except the isolated populations 11 from Tozeur and 12 from Selja), the results were much less bulky compared to the complete data set (Table 3): While MCHEZA found 11 loci falling outside a 99% confidence interval for neutral loci, five of these could be also corroborated as being under selection in the BayeScan analysis. Of these, three loci (L039, L040, L459) were also found with band presence/absence being significantly correlated with at least one bioclimatic factor.

**Table 3:** Characterization of 12 outlier loci found as being under selection by one (italics), two (simple), or three (bold) of the three methods implemented in MCHEZA, BayeScan, and Samβada, respectively, in the reduced data set (pop01‐pop10). For each locus, estimates of *F*_ST_ and associated significance values (false discovery rate FDR) obtained using MCHEZA and BayeScan are given. Outlier loci found by Samβada are documented together with the environmental variable(s) showing significant correlations with the presence of the dominant allele

	Locus	MCHEZA		BayeScan		Samβada
		*F*_ST_	FDR	*F*_ST_	*q*‐value	
**D2**	**L039**	**0.164**	**<0.01**	**0.090**	**0.0179**	*bio18*
**D2**	**L040**	**0.193**	**<0.005**	**0.128**	**0.0005**	*PC3, bio18*
D2	L139	**0.180**	**<0.005**	**0.079**	**0.0477**	
*D3*	*L346*	**0.175**	**<0.005**	0.054	0.1134	
*D3*	*L452*	**0.146**	**<0.01**	0.038	0.2279	
*D3*	*L457*	**0.136**	**<0.01**	0.027	0.4750	
**D3**	**L459**	**0.309**	**<0.005**	**0.239**	**<0.0001**	*bio05, bio12, bio13, bio18*
*D3*	*L461*	**0.081**	**<0.05**	0.029	0.4238	
*D3*	*L480*	**0.129**	**<0.01**	0.027	0.4968	
*D4*	*L514*	0.083	>0.05	**0.088**	**0.0354**	
D4	L548	**0.198**	**<0.005**	**0.108**	**0.0043**	
*D4*	*L692*	**0.142**	**<0.01**	0.025	0.5164	

### 3.5 Relationship between genetic and phenotypic divergence

Tables 4 and 5 summarize the results of the Generalized Analysis of Molecular Variance (GAMOVA) for the complete (12 populations) and the reduced data set (10 populations), respectively. In the complete data set (Table 4), two morphological characters (i.e., *V02*: absolute area of the sixth cauline leaf; *V15*: leaf shape of the sixth cauline leaf, principal component axis PC1) were found being significantly correlated with the set of outlier loci. This result was observed irrespective of the number of outlier loci and their composition in the three different sets that were found using three different methods for detection of AFLP loci under selection. However, the same characters were also found correlating significantly with the sets of the remaining, neutral loci in all cases. In addition to that, further three characters (i.e., *V04*: number of flowers; *V07*: number of leaves; *V17*: leaf shape of the sixth cauline leaf, principal component axis PC3) showed significant correlations with population differentiation patterns in the sets of neutral loci.

**Table 4:** Results of the GAMOVA (Generalized Analysis of Molecular Variance; Nievergelt et al., 2007) tests conducted in the complete data set (pop01‐pop12) of the present study. For each of the 17 variables (*V01–V10*: morphological and life‐history traits, see Table S2; *V11–V14*: principal component coordinates PC 1 to PC 4 of flower morphometrics, see Figure 2; *V15–V17*: principal component coordinates PC 1 to PC 3 of leaf morphometrics, see Figure 1), significant correlations with outlier and neutral locus sets resulting from one (Samβada), two (MCHEZA, BayeScan), and three (MCHEZA ∩ BayeScan ∩ Samβada) analyses for loci under selection are given in bold. Additionally, for each locus set, the results of a Mantel (Mantel, 1967) test for correlation between pairwise geographical distances and pairwise F_ST_ values among populations are given

	Populations 1–12											
	MCHEZA ∩ BayeScan				MCHEZA ∩ BayeScan ∩ Samβada				Samβada			
	Outlier loci (*N *= 16)		Neutral loci (*N *= 716)		Outlier loci (*N *= 12)		Neutral loci (*N *= 720)		Outlier loci (*N *= 19)		Neutral loci (*N *= 713)	
	Pseudo‐*F*	*p‐*Value	Pseudo‐*F*	*p‐*Value	Pseudo‐*F*	*p‐*Value	Pseudo‐*F*	*p‐*Value	Pseudo‐*F*	*p‐*Value	Pseudo‐*F*	*p‐*Value
*V01*	1.40	.261	2.35	.135	0.91	.428	2.56	.121	1.04	.364	2.69	.112
*V02*	**7.01**	**.004**	**11.39**	**.002**	**5.93**	**.012**	**11.70**	**.002**	**6.15**	**.012**	**11.95**	**.002**
*V03*	0.31	.782	0.50	.601	0.29	.776	0.45	.620	0.19	.842	0.55	.545
*V04*	2.03	.122	**7.50**	**.008**	1.37	.258	**7.60**	**.009**	1.54	.203	**8.35**	**.005**
*V05*	2.05	.173	0.26	.776	2.16	.168	0.30	.722	1.99	.189	0.24	.775
*V06*	0.36	.747	0.55	.576	0.34	.734	0.58	.543	0.48	.634	0.59	.542
*V07*	2.17	.155	**4.80**	**.038**	1.47	.260	**5.14**	**.031**	1.39	.272	**5.69**	**.026**
*V08*	0.82	.466	1.88	.180	0.95	.405	1.83	.191	0.88	.418	1.98	.172
*V09*	1.02	.372	2.47	.121	0.96	.397	2.38	.130	1.18	.311	2.38	.135
*V10*	1.49	.213	1.58	.222	1.65	.195	1.50	.240	1.54	.206	1.52	.244
*V11*	–0.10	.994	0.06	.918	–0.14	.996	0.03	.927	–0.09	.995	0.03	.922
*V12*	2.42	.112	2.83	.092	1.99	.158	3.04	.084	2.43	.116	2.79	.100
*V13*	1.21	.312	3.31	.073	1.22	.315	3.06	.087	1.13	.320	3.34	.072
*V14*	0.21	.818	0.83	.409	0.15	.853	0.79	.420	0.25	.770	0.83	.404
*V15*	**4.92**	**.015**	**7.14**	**.009**	**4.48**	**.030**	**7.16**	**.012**	**4.58**	**.034**	**7.08**	**.010**
*V16*	1.25	.305	1.16	.308	1.19	.337	1.22	.291	1.16	.321	1.24	.282
*V17*	3.96	.054	**12.45**	**.002**	2.79	.106	**13.16**	**.001**	2.77	.102	**15.11**	**.001**

Mantel tests												
	*r*_*xy*_	*p‐*Value	*r*_*xy*_	*p‐*Value	*r*_*xy*_	*p‐*Value	*r*_*xy*_	*p‐*Value	*r*_*xy*_	*p‐*Value	*r*_*xy*_	*p‐*Value
	**0.870**	**<.001**	**0.731**	**<.001**	**0.869**	**<.001**	**0.723**	**<.001**	**0.904**	**<.001**	**0.662**	**<.001**

**Table 5:** Results of the GAMOVA (Generalized Analysis of Molecular Variance; Nievergelt et al., 2007) tests conducted in the resulted data set (pop01–pop10) of the present study. For each of the 17 variables (*V01–V10*: morphological and life‐history traits, see Table S2; *V11–V14*: principal component coordinates PC 1 to PC 4 of flower morphometrics, see Figure 2; *V15–V17*: principal component coordinates PC 1 to PC 3 of leaf morphometrics, see Figure 1), significant correlations with outlier and neutral locus sets resulting from one (Samβada), two (MCHEZA, BayeScan), and three (MCHEZA ∩ BayeScan ∩ Samβada) analyses for loci under selection are given in bold. Additionally, for each locus set, the results of a Mantel (Mantel, 1967) test for correlation between pairwise geographical distances and pairwise F_ST_ values among populations are given

	Populations 1–10							
	MCHEZA ∩ BayeScan				MCHEZA ∩ BayeScan ∩ Samβada			
	Outlier loci (*N *= 5)		Neutral loci (*N *= 727)		Outlier loci (*N *= 3)		Neutral loci (*N *= 729)	
	Pseudo‐*F*	*p‐*Value	Pseudo‐*F*	*p‐*Value	Pseudo‐*F*	*p‐*Value	Pseudo‐*F*	*p‐*Value
V01	1.15	.366	2.67	.140	0.20	.704	2.97	.126
V02	1.10	.372	**4.31**	**.041**	0.42	.571	**4.53**	**.045**
V03	–0.23	.990	0.64	.438	–0.29	.996	0.56	.471
V04	0.96	.432	3.45	.088	0.21	.705	3.58	.092
V05	1.89	.203	2.25	.156	1.43	.277	2.36	.171
V06	0.59	.575	0.50	.604	0.60	.479	0.53	.559
V07	**4.65**	**.045**	**8.56**	**.016**	2.98	.125	**9.15**	**.021**
V08	–0.01	.885	0.31	.723	0.33	.611	0.34	.669
V09	0.17	.740	0.74	.426	0.09	.800	0.70	.419
V10	0.15	.752	0.12	.791	–0.07	.924	0.13	.761
V11	0.19	.750	0.02	.917	0.20	.703	–0.04	.932
V12	1.25	.338	1.28	.288	0.98	.364	1.37	.279
V13	0.61	.567	0.62	.547	0.83	.407	0.42	.646
V14	0.11	.783	0.28	.711	0.20	.698	0.26	.700
V15	3.62	.066	1.48	.264	**6.66**	**.038**	1.23	.324
V16	2.66	.114	2.35	.162	1.26	.304	2.64	.140
V17	3.28	.091	**10.54**	**.015**	1.46	.283	**11.37**	**.017**

Mantel tests								
	*r*_*xy*_	*p‐*Value	*r*_*xy*_	*p‐*Value	*r*_*xy*_	*p‐*Value	*r*_*xy*_	*p‐*Value
	**0.552**	**.001**	**0.510**	**.017**	**0.389**	**.015**	**0.524**	**.009**

When the search for loci under selection was reduced to differentiation patterns among the 10 SE Tunisian populations (populations 1–10; Table 5), correlations between morphological characters and sets of selected loci gained significance only in two cases: (1) character *V07* (number of leaves) in the set of outlier loci found with Mcheza and BayeScan (*n *= 5 loci) and (2) character *V15* (leaf shape of the sixth cauline leaf, principal component axis PC1) in the intersecting set of outlier loci found with Mcheza, BayeScan, and Samβada (*n *= 3 loci). While in the former case (*V07*), a significant correlation between character expression and population differentiation patterns was also observed for the set of neutral loci (*n *= 727 loci), this correlation lacks between character *V15* and the set of neutral loci (*n *= 729) in the latter one. Additionally and in correspondence with the results of the 12‐populations data set, characters *V02* (absolute area of the sixth cauline leaf) and *V17* (leaf shape of the sixth cauline leaf, principal component axis PC3) were also found being significantly correlated with the set of neutral loci.

**Figure 4:**
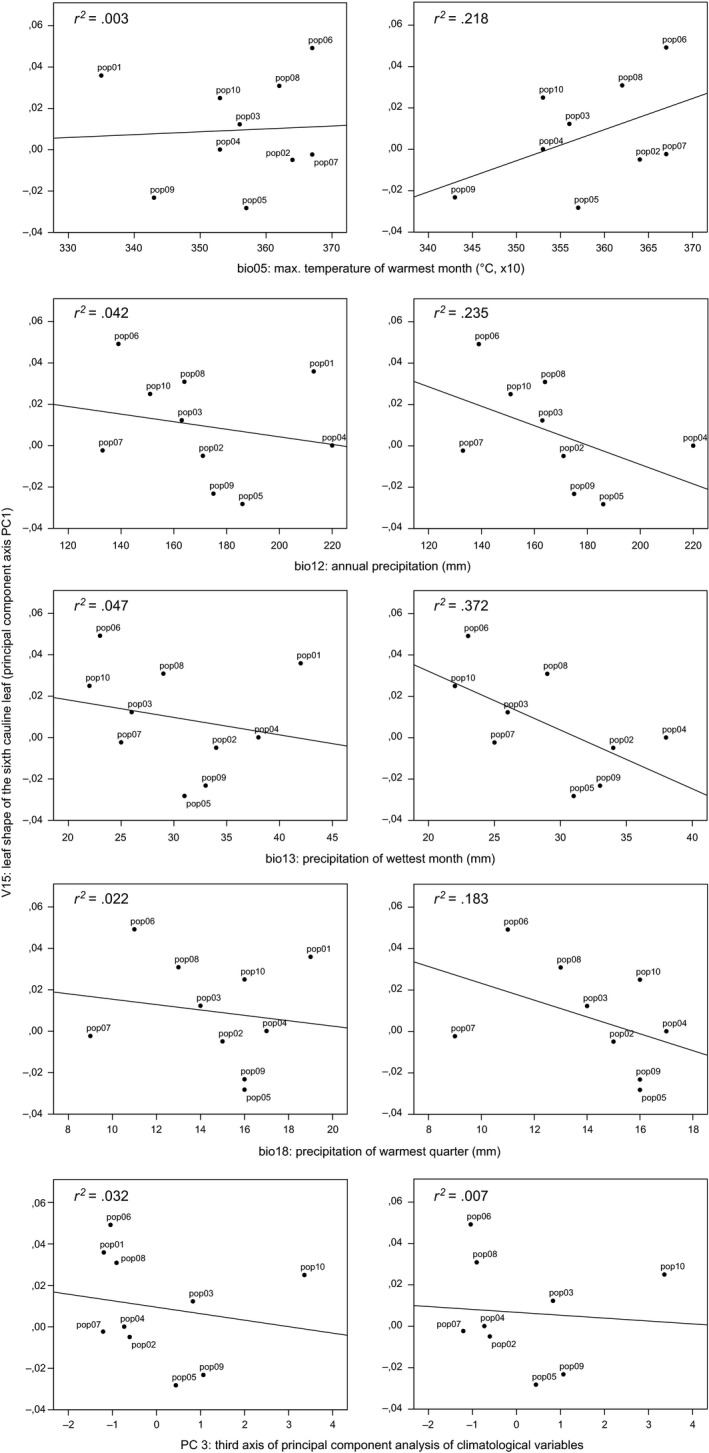
Linear regression of trait *V15* (leaf shape of the sixth cauline leaf described by PC 1 of the morphometric analysis; see Figure 2) on four individual bioclimatic variables and the third axis (PC 3) of a principal component analysis (PCA) of all 20 bioclimatological variables (Table S3). In the left column, all ten populations (pop1–pop10) of the reduced data set are used, while in the right column, pop1 (Djerba) has been omitted, leading to a higher coefficient of determination *r* ^*2*^ in the case of the four individual bioclimatic variables *bio05, bio12, bio13,* and *bio18*

Following the reasoning behind our search strategy for life‐history and morphological traits under selection (Table 1), *V15* represents the only trait, for which neutral variation could be ruled out significantly. Regressing of population means of *V15* on the bioclimatic variables with a significant contribution to the selection regime of the reduced data set (i.e., *bio05, bio12, bio13, bio18, PC3;* Table S3), however, revealed quite spurious relationships (Figure 4, left). The situation improved considerably after removing population 1 (Djerba) and revealed marked trends for the relationships of *V15* (leaf shape) with *bio05* (max. temperature of warmest month), *bio12* (annual precipitation), *bio13* (precipitation in wettest month), and *bio18* (precipitation in warmest quarter; Figure 4, right). Deducing from these trends, narrow leaves were found, therefore, in plants from populations with lower temperatures in the warmest month (*bio05*) and higher annual and seasonal precipitation values (*bio12, bio13, bio18*).

### 3.6 Correlation of selected and neutral loci with geography

Tables 4 and 5 also summarize the results of Mantel test for correlation between genetic and geographical distances for all non‐overlapping subsets of loci (“selected” vs. “neutral” ones) according to the results of the MCHEZA, BayeScan, and Samβada analyses. For all subsets, irrespective of being sets of selected or neutral loci and irrespective of being based on all 12 populations or only populations 1–10, a significant correlation between population structure and geographical distances was found. Correlation coefficients *r*_*xy*_ gained higher values (*r*_*xy*_ = 0.869–0.904 for selected and *r*_*xy*_ = 0.662–0.731 for neutral loci) when populations included in the Mantel tests comprised also the genetically deviating and geographically more remote populations 11 (Tozeur) and 12 (Selja; Table 4), while the geographical signal was found being weaker (*r*_*xy*_ = 0.389–0.552 for selected and *r*_*xy*_ = 0.510–0.524 for neutral loci) when these populations were excluded from the analyses (Table 5).

## 4 DISCUSSION

### 4.1 Population structure and taxonomic implications

Our AFLP‐based analysis of the population structure of *Diplotaxis harra* in central and south Tunisia revealed a clear picture of genetic bipartition, with populations 1–9 (SE Tunisia) on the one and populations 11 (Tozeur) and 12 (Selja) on the other hand, while population 10 (El Hamma) is found being both geographically and genetically intermediate between these two groups. This discontinuous population genetic pattern was also found being paralleled to some degree by the analysis of leaf shapes, where plants from populations 11 and 12 exhibited a tendency toward somewhat narrower leaves than plants from other populations under study, however, with a broad range of overlap in this feature (Figure S2).

Following Pottier‐Alapetite (1979) and the Euro+Med plantbase (Euro+Med 2006), *D. harra* is represented in Tunisia by two subspecies, *D. harra* subsp. *harra* and subsp. *crassifolia* (Raf.) Maire. As Pottier‐Alapetite (1979) indicates the latter subspecies for the “Gorges des Seldja” while many of her indications for the former subspecies (e.g., “Gafsa, Matmatas”) rather correspond to localities sampled for the present study under population numbers 7, 8, and 10, it may appear self‐evident that the genetic (and morphological) bipartition observed might be caused by sampling of the two subspecies and might indicate rather a taxonomic‐biogeographical structure than a selection‐dependent and taxonomy‐independent one. There are, however, considerable arguments against this interpretation: (1) in contrast to the above‐mentioned two sources, Maire (1965) in his treatment of the genus in the *Flore d′Afrique du Nord* only indicates the first subspecies, *D. harra* subsp. *harra,* for Tunisia, but not the second one (only found in Morocco and Algeria); (2) according to diagnostic differences between the two subspecies given by Fennane (1999), Maire (1965), and Pottier‐Alapetite (1979) with leaves of subsp. *harra* being furnished with 5–15 teeth along each side of the leaves and only 1–6 teeth in subsp. *crassifolia,* all surveyed populations corresponded to the former type; and (3) while the life‐form of subsp. *harra* is described as being annual to shortly perennial by Pottier‐Alapetite (1979), subsp. *crassifolia* is described as being strictly perennial and subshrubby, a feature definitively not exhibited by the plants of the present study, which died at the end of the fruiting period even under optimal glasshouse/garden conditions. This is additionally corroborated by observations of *Diplotaxis harra* (subsp. *harra*) in Egypt done by Hegazy (2001) who describes that a part of populations of this species exhibits coppiced and perennial behavior. Therefore, we are quite confident that the results of our selection study are not invalidated by a background taxonomical structure and infraspecific biogeographical or neutral demographic history (phylogeographical) patterns. Nevertheless, in order to safeguard our results against any possible taxonomy‐prone distortions (and possible biases caused by the artificial geographical sampling gap between populations 1–10 and populations 11–12), we did all of our analyses both in the full (populations 1–12) and in a reduced data set (populations 1–10).

### 4.2 Detecting selection in SE Tunisian *Diplotaxis harra* populations

In both data sets, the complete one with populations 1–12 and the reduced one excluding populations 11 and 12, we found a good, yet not complete correspondence between the two *F*_ST_‐based methods for detection of loci under selection. Correspondence was found being higher in the complete data set (with 16 of 23 loci: 70%) than in the reduced one (five of 12 loci: 42%), as was the absolute number of loci identified as being under selection with both methods (16 vs. 5). Therefore, in relation to the total number of AFLP loci screened (732), our conservative approach of only identifying outliers as loci detected by all three independent tests revealed only 1.6% (complete data set) or even only 0.4% (reduced data set) of all loci as being under selection. These percentages considerably fall below the values reported by Nosil, Funk, and Ortiz‐Barrientos (2009) for AFLP‐based genomic scans using Dfdist/MCHEZA (5–10%) and they are far from the 8.9% of loci found being under precipitation‐related environmental selection in a comparable study of *Geropogon hybridus* (L.) Sch.Bip. (Compositae) populations in the southern Judea Lowland (Israel; Müller et al., 2017). One explanation for the low number of candidate loci found in our study may be its small geographical scale with surveyed populations separated by only 20.4 km (populations 3 and 4) to 126.8 km (populations 6 and 10; reduced data set) or 256.1 km (populations 6 and 12; complete data set) when compared to studies carried out at both regional or even continental scales (Manel et al., 2010). Another explanation, however, may be that our conservative approach for outlier detection may have led to an inflated type II error and an underestimation of the number of loci under selection in our study compared to other ones. However, following the argumentation of Henry and Russello (2013), we consider the applied cross‐validation procedure a valid strategy for increasing confidence in identified outliers.

We found that inclusion of the two geographically, climatically, and genetically deviating populations from the west of Chott el Djerid was tremendously influencing the number of outlier loci both detected by the two *F*_ST_‐based and the environment‐allele association approaches. The Samβada results (Table 2) demonstrate that divergent selection at 19 of 26 outlier loci is caused by climatological variables, either by individual ones (e.g., *bio15,* precipitation seasonality), but mostly by conglomerates of many, covarying ones and their surrogates PC1 and PC3 of the principal component analysis (PCA). Interestingly, almost all climatological variables with high factor loadings on PC2 (i.e., *bio01, bio03, bio06, bio11, bio14,* and *bio20;* see Table S4), which is the principal component axis that mostly differentiates among populations 1–10, are excluded from the list of variables with a significant environment‐allele association in the analysis of the complete data set. As a consequence, climate‐associated selection along the small‐scale gradient between the Mediterranean conditions of Djerba on the one (populations 1) and steppe habitats of inland populations in the Matmata Mts. (populations 4, 5, and 8) is eclipsed by the larger‐scale patterns of environment‐mediated divergent selection between *Diplotaxis harra* populations in proximity of the Mediterranean Sea (populations 1–10) and the semidesert habitats west of Chott el Djerid (populations 11 and 12). However, as isolation‐by‐distance (IBD) tests based on the different sets of neutral loci always received highly significant support (Table 4), we cannot preclude completely that this larger‐scale pattern may be influenced by demography and neutral history of populations (phylogeography) in the background of natural selection.

When the two deviating semidesert populations are excluded, environmental gradients get much shorter but exhibit a more continuous pattern of variation among the ten remaining habitats surveyed (as demonstrated by the PCA in Figure S6), allowing for a possibly less phylogeographically biased analysis of molecular and trait selection. This expectation is supported by Lotterhos & Whitlock (2015) who have hypothesized that local adaptation may be most informatively studied by outlier detection methods when ecologically divergent populations sampled are geographically adjacent (and therefore genetically similar for neutral genes). As expected, the search for candidate loci resulted in a smaller number of loci under selection, five being jointly detected by the two *F*_ST_‐based methods and only three of these corroborated by the environment‐allele association approach (Table 3). The climate variables responsible for this small‐scale selection were identified as being connected to precipitation (*bio12*: annual precipitation; *bio13*: precipitation in wettest month; *bio18*: precipitation in the warmest quarter), temperature (*bio05*: maximum temperature in the warmest month), and the climatic surrogate variable of *PC3* (being mainly under the influence of *latitude, longitude,* and *bio15*: precipitation seasonality); all of them fitting the steep gradient between Mediterranean and steppe vegetation described by Frankenberg and Klaus (1987) for the study area.

### 4.3 Traits under climate‐dependent selection in SE Tunisian *Diplotaxis harra* populations

Irrespective of the composition of the set of outlier loci found with the three detection methods applied, the GAMOVA analyses of the complete data set revealed statistically significant correlations between divergent selection patterns and two morphological traits (Table 4): *V02* (accounting for the absolute size of the sixth leaf produced along the main shoot) and *V15* (length‐width ratio of the lamina of the sixth leaf). However, as both traits (along with three others, *V04*: number of flowers along main shoot; *V07*: number of leaves along main axis; *V17*: pointedness of lamina of sixth leaf) also show strong correlations with the set of neutrally evolving loci, phenotypic divergence among populations could be caused by both selection *and* neutral processes, and the latter could not be excluded with confidence as explanation (see Table 1). Extremely strong isolation‐by‐distance (IBD) signals for both outlier and neutral loci sets corroborate the interpretation of morphological traits showing variation rather caused by neutral biogeographical and/or random factors than by environmentally mediated divergent selection.

The picture is slightly different in the small‐scale analysis with the reduced data set of populations 1–10 (Table 5). In the outlier loci set found with the two *F*_ST_‐based methods, only *V07* (number of leaves) was detected as being under divergent selection; however, as in the complete data set, this morphological character showed an equally strong correlation with the set of neutrally evolving markers and is interpreted here again as possibly caused by random or population history factors. When the number of outlier loci is reduced to three by additionally accounting for the results of the Samβada analysis; however, *V15* (describing the shape of leaves in the Fourier outline analysis) displays a significant correlation with the outlier loci set, which is not paralleled by a correlation with the neutral loci set. As the outlier loci also exhibit a significant IBD signal, this indicates that leaf shape and especially the width‐length ratio determining the first principal component of the Fourier analysis (PC1: *V15*) is under a climate‐mediated divergent selection along a geographical cline (see Table 1). Following studies addressing the genetic background of leaf‐length/width ratio (leaf index) in the crucifer *Arabidopsis thaliana* (Horiguchi, Fujikura, Ishikawa, & Tsukaya, 2006; Tsukaya, 2006), the leaf index is controlled by four regulating systems being the combination of genes influencing the polar cell elongation (cell shape) or cell proliferation (cell number) in either the leaf‐length or the leaf‐width direction. Therefore, one could legitimately assume that this important functional trait is under oligogenic control also in the related crucifer *Diplotaxis harra* and that the three outlier loci (L039, L040, and L459) found could be linked with at least one of these genes. Methods described by Paris et al. (2012) could be used in the future to gain nucleotide sequences of these AFLP loci of interest and test their identity with length/width ratio genes of *Arabidopsis*.

The correlational trends in leaf shape as a morphological trait under environmental selection, with broader leaves being observed in plants from semidesert populations with higher temperatures in the warmest month (*bio05*) and lower annual and seasonal precipitation values (*bio12, bio13, bio18*)*,* appears being counter‐intuitive because xeromorphic leaves are usually smaller and narrower than leaves from more humid environments. This tendency has been observed, for example, in leaves of summer ecotypes vs. leaves of winter ecotypes in the Sicilian *Diplotaxis erucoides* (Schleser, Bernhardt, & Hurka, 1989). Additionally, among other traits, length‐width ratio of leaves was found exhibiting considerable plasticity in *Piriqueta caroliniana* Urban (Turneraceae), with narrower leaves being produced under more arid conditions in successive years (Picotte, Rhode, & Cruzan, 2009) or even in successive seasons of the same year (Picotte et al. 2007). However, as we lack measurements from natural habitats and our experiment has not been carried out under natural conditions in common gardens with Mediterranean and/or semidesert climate but under glasshouse/garden conditions with optimal supplies of water, light, and nutrients, the observed trends rather demonstrate that plants with provenances from semi‐arid habitats either *have* broader leaves or are capable to produce broader leaves than those from more mesic conditions when confronted with optimal growth conditions and that this trait or the plasticity of this trait is selected for. As described by Nicotra et al. (2011: 542), the functional significance of leaf shape may lie in its influence on leaf thermal regulation and its hydraulic properties, the latter “likely to be of greater importance.”

While it is easily conceivable that more unpredictable precipitation at semi‐arid inland habitats may select for a higher plasticity in leaf shape than the more constant conditions along the Mediterranean coast, allowing plants to adjust to highly temporally variable aspects of transpirational demand (Sultan, 2000), our present observations could only be the starting point for more comprehensive, hypothesis‐driven experiments addressing the evolutionary significance of this functional trait and its plasticity. Common‐garden cultivation under different watering regimes (genotype‐by‐environment interaction) should be the first experiments in that direction (Des Marais, Hernandez, & Juenger, 2013), followed by artificial selection experiments and experiments addressing the question whether leaf‐shape plasticity is adaptive (Scheiner, 1993).

## CONFLICT OF INTEREST

None declared.
