# Supplementary figures and images for: Are there morphological and life‐history traits under climate‐dependent differential selection in S Tunesian Diplotaxis harra (Forssk.) Boiss. (Brassicaceae) populations?
Source: Ecol Evol. 2017 Dec 15;8(2):1047–62. doi: 10.1002/ece3.3705 (PMC5773308; doi:10.1002/ece3.3705)

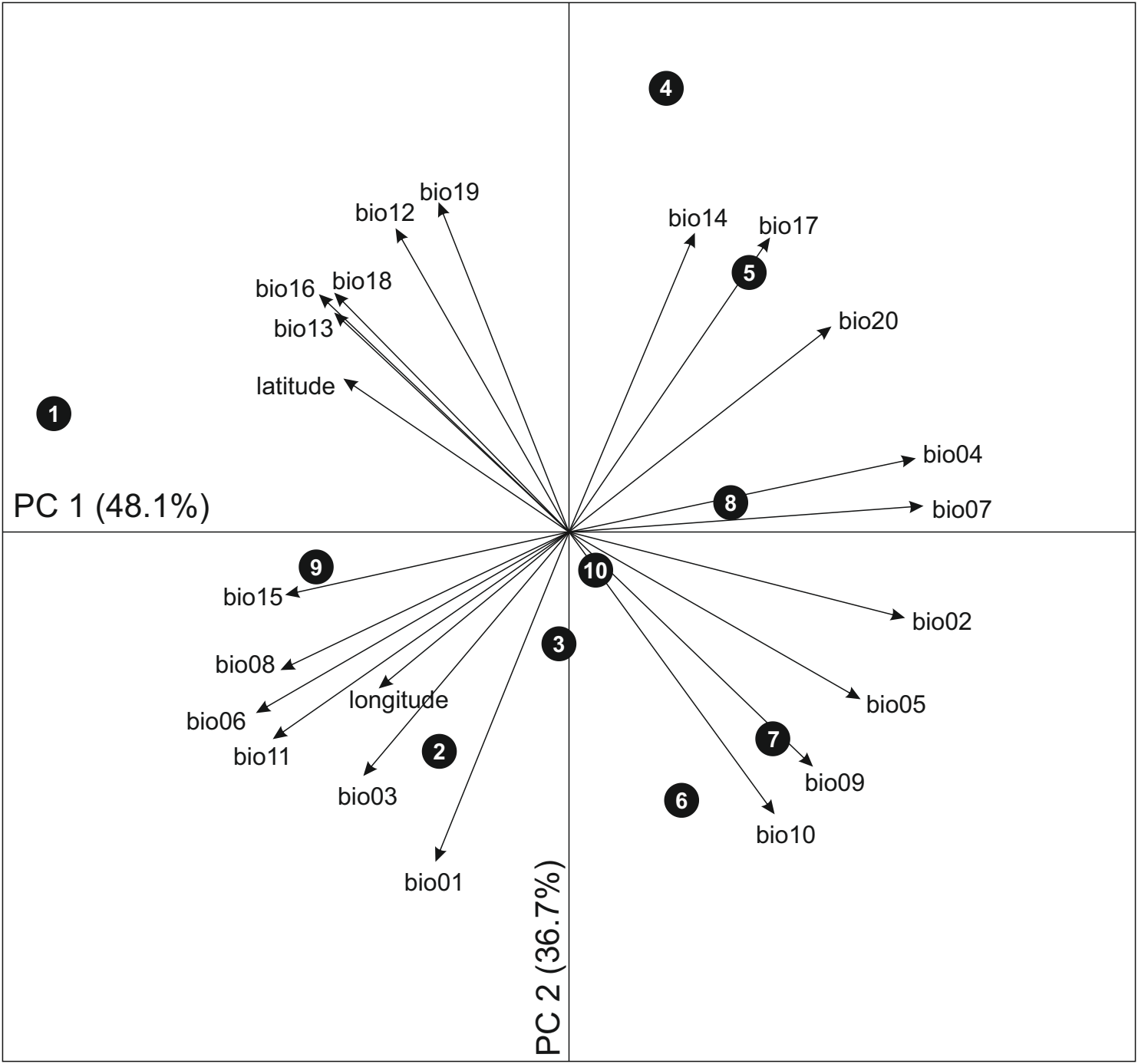

Supplement: Supplementary file 6 [file ECE3-8-1047-s006.pdf]

# Fst/He

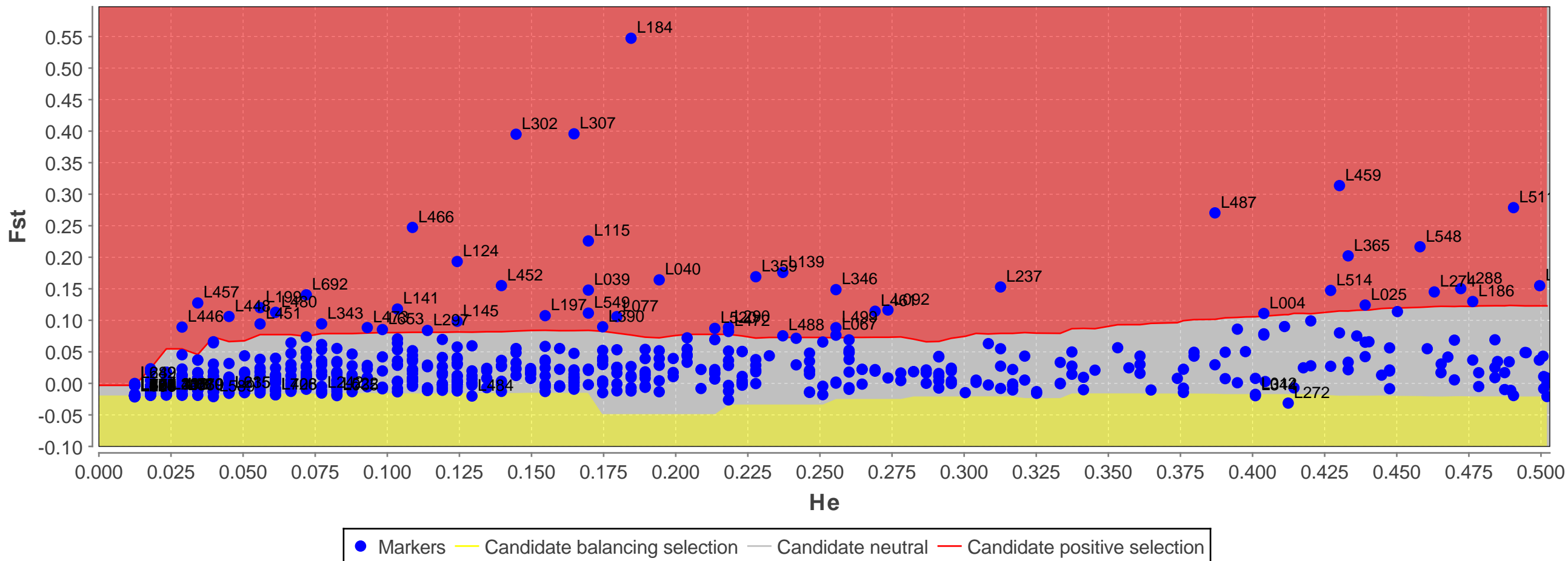

Supplement: Supplementary file 7 [file ECE3-8-1047-s007.pdf]
