# Supplementary material for: Are there morphological and life‐history traits under climate‐dependent differential selection in S Tunesian Diplotaxis harra (Forssk.) Boiss. (Brassicaceae) populations?
Source: Ecol Evol. 2017 Dec 15;8(2):1047–62. doi: 10.1002/ece3.3705 (PMC5773308; doi:10.1002/ece3.3705)
Supplement: Supplementary file 8 [file ECE3-8-1047-s008.doc]

**Table S1.** Populations of *Diplotaxis harra* studied in the present study, with geographical coordinates, detailed information on sample localities and voucher specimens, and sample size *(n)* for each population.

| **pop** | **locality** | **coordinates** | **voucher specimen** | **n** |
| --- | --- | --- | --- | --- |
| **Dh01** | Djerba | 33 46 14.6 N 10 48 54.5 E | Gov. Medenine, Djerba, May - Adjim, 6 km SW May, 16 m, 20.03.2009, *Vogt 16552, Oberprieler 10495 & Gstöttl* (B 10 0216469) | 15 |
| **Dh02** | Bou Grara | 33 27 16.5 N 10 35 44.5 E | Gov. Medenine, Bou Grara - Medenine, 12 km SW Bou Grara, 21 m, 21.03.2009, *Vogt 16539, Oberprieler 10482 & Gstöttl* (B 10 0216459) | 15 |
| **Dh03** | Amra | 33 24 53.9 N 10 15 55.7 E | Gov. Medenine, Medenine - Matmata, 5 km W junction with road C114, 170 m, 21.03.2009, *Vogt 16542, Oberprieler 10485 & Gstöttl* (B 10 0216460) | 15 |
| **Dh04** | Toujane | 33 29 08.6 N 10 03 36.2 E | Gov. Gabès, Medenine - Matmata, 10 km WNW Toujane, 469 m, 21.03.2009, *Vogt 16546, Oberprieler 10489 & Gstöttl* (B 10 0216462) | 16 |
| **Dh05** | Tamasrat | 33 32 00.4 N 09 49 21.0 E | Gov. Gabès, Matmata, 3 km W Tamasrat, 351 m, 21.03.2009, *Vogt 16551, Oberprieler 19494 & Gstöttl* (B 10 0216461) | 12 |
| **Dh06** | Kasba Drina | 33 01 26.1 N 10 36 29.0 E | Gov. Tataouine, 6 km NO junction with C111, 133 m, 22.03.2009, *Vogt 16553, Oberprieler 10496 & Gstöttl* (B 10 0216470) | 15 |
| **Dh07** | Tataouine | 32 55 22.5 N 10 23 51.2 E | Gov. Tataouine, Tataouine - Ghomrassen, 5 km NW Tataouine, 275 m, 22.03.2009, *Vogt 16565, Oberprieler 10508 & Gstöttl* (B 10 0216474) | 15 |
| **Dh08** | Ksar Hadada | 33 08 35.2 N 10 17 29.7 E | Gov. Tataouine, Ghomrassen - Beni Kheddache, 5 km N Ksar Hadada, 398 m, 22.03.2009, *Vogt 16569, Oberprieler 10512 & Gstöttl* (B 10 0216468) | 14 |
| **Dh09** | Mareth | 33 39 13.4 N 10 16 34.3 E | Gov. Gabès, Medenine - Gabès, 4 km N Mareth, 33 m, 23.03.2009, *Vogt 16574, Oberprieler 10517 & Gstöttl* (B 10 0216477) | 19 |
| **Dh10** | Sambar | 33 53 12.1 N 09 43 01.4 E | Gov. Gabès, Gabès - Kebili, 4 km W Sambar, 72 m, 23.03.2009, *Vogt 16579, Oberprieler 10522 & Gstöttl* (B 10 0216553) | 15 |
| **Dh11** | Tozeur | 34 03 12.7 N 08 14 10.5 E | Gov. Tozeur, Kariz, 65 m, 23.03.2009, *Vogt 16591, Oberprieler 10534 & Gstöttl* (B 10 0216476) | 15 |
| **Dh12** | Selja | 34 20 08.3 N 08 20 12.3 E | Gov. Gafsa, Metlaoui, Selja gorge, 210-250 m, 23.03.2009, no voucher specimen. | 16 |
