# Supplementary material for: Are there morphological and life‐history traits under climate‐dependent differential selection in S Tunesian Diplotaxis harra (Forssk.) Boiss. (Brassicaceae) populations?
Source: Ecol Evol. 2017 Dec 15;8(2):1047–62. doi: 10.1002/ece3.3705 (PMC5773308; doi:10.1002/ece3.3705)
Supplement: Supplementary file 9 [file ECE3-8-1047-s009.doc]

**Table S2.** Morphological and life-history traits of *Diplotaxis harra* surveyed in common-garden experiments of the present study. For each population, the number of individuals measured *(n)* and arithmetic means (standard deviation) of the ten traits are given.

| **variable** |  | **population** | | | | | | | | | | | |
| --- | --- | --- | --- | --- | --- | --- | --- | --- | --- | --- | --- | --- | --- |
|  |  | **pop01** | **pop02** | **pop03** | **pop04** | **pop05** | **pop06** | **pop07** | **pop08** | **pop09** | **pop10** | **pop11** | **pop12** |
| *n* |  | 15 | 15 | 15 | 16 | 16 | 15 | 14 | 15 | 19 | 15 | 15 | 15 |
| *V01* | time between sowing (March 25) and the opening of the first flower (days) | 52.3 (4.0) | 63.3 (10.9) | 61.7 (3.1) | 62.6 (5.3) | 65.9 (8.4) | 67.0 (6.3) | 61.7 (8.0) | 64.2 (5.4) | 61.7 (8.3) | 56.0 (5.9) | 56.9 (6.3) | 61.9 (6.9) |
| *V02* | absolute area of leaf lamina of sixth cauline leaf (cm^2^) | 22.7 (7.0) | 25.2 (10.8) | 27.7 (6.8) | 23.1 (7.5) | 24.5 (9.8) | 29.4 (9.4) | 26.4 (10.9) | 23.0 (9.1) | 28.4 (7.6) | 24.3 (8.7) | 19.3 (5.6) | 19.9 (10.6) |
| *V03* | time between starting of flowering and formation of first ripe fruits (days) | 49.1 (7.8) | 43.7 (10.6) | 41.3 (5.1) | 45.1 (5.8) | 38.4 (7.8) | 41.3 (4.3) | 42.4 (7.0) | 42.3 (6.1) | 41.4 (7.8) | 41.6 (5.6) | 48.5 (10.7) | 40.5 (4.5) |
| *V04* | number of flowers along main shoot | 81.4 (28.1) | 66.9 (32.1) | 93.2 (21.8) | 69.0 (29.9) | 63.1 (28.5) | 91.5 (23.0) | 60.9 (40.3) | 65.6 (36.4) | 72.6 (41.1) | 70.7 (28.2) | 55.9 (21.3) | 64.4 (17.5) |
| *V05* | flower size (mean length of four petals) | 8.3 (1.4) | 8.4 (1.2) | 9.5 (1.2) | 9.2 (1.3) | 9.8 (1.2) | 8.1 (1.1) | 9.5 (1.4) | 9.1 (1.5) | 9.6 (1.2) | 9.2 (1.2) | 8.4 (1.1) | 8.4 (1.2) |
| *V06* | average number of seeds per fruit (mean of 3 siliques) | 177 (26) | 165 (23) | 192 (19) | 190 (25) | 174 (20) | 178 (25) | 165 (37) | 167 (23) | 175 (32) | 190 (21) | 177 (31) | 165 (24) |
| *V07* | number of leaves along main shoot | 9.4 (1.1) | 11.1 (1.4) | 12.2 (1.6) | 12.3 (1.8) | 12.9 (2.5) | 15.9 (2.6) | 13.4 (3.1) | 13.5 (2.4) | 13.0 (1.5) | 10.5 (1.6) | 11.5 (2.1) | 12.0 (2.8) |
| *V08* | number of hairs along lateral leaf margins (5 mm, sixth cauline leaf) | 7.0 (2.0) | 7.6 (3.1) | 10.0 (2.5) | 9.4 (3.2) | 6.5 (3.0) | 5.4 (2.0) | 10.9 (4.4) | 11.9 (4.3) | 7.8 (2.5) | 8.9 (3.5) | 9.9 (4.1) | 10.5 (4.6) |
| *V09* | total length of main shoot (cm) | 115 (25) | 82 (24) | 103 (20) | 84 (20) | 80 (25) | 93 (11) | 87 (31) | 78 (25) | 87 (26) | 86 (21) | 69 (17) | 83 (19) |
| *V10* | number of side shoots | 4.2 (2.8) | 9.3 (8.5) | 4.5 (3.6) | 5.4 (6.0) | 4.4 (3.3) | 5.1 (3.1) | 4.9 (4.0) | 7.6 (5.8) | 5.5 (3.1) | 4.2 (2.4) | 9.0 (4.6) | 5.9 (4.8) |
