# Supplementary material for: Are there morphological and life‐history traits under climate‐dependent differential selection in S Tunesian Diplotaxis harra (Forssk.) Boiss. (Brassicaceae) populations?
Source: Ecol Evol. 2017 Dec 15;8(2):1047–62. doi: 10.1002/ece3.3705 (PMC5773308; doi:10.1002/ece3.3705)
Supplement: Supplementary file 10 [file ECE3-8-1047-s010.doc]

**Table S3.** Bioclimatological variables used to characterise the S Tunisian populations of *Diplotaxis harra* used in the present study. Additionally, the number of candidate loci, for which a Samβada (Joost et al. 2007, Stucki et al. 2016) analysis gave significant results in the complete (pop01-pop12) and the reduced (pop01-pop10) data set, respectively, are given.

| **variable** | **definition, unit** | **number of Samβada loci** | |
| --- | --- | --- | --- |
|  |  | **populations 1-12** | **populations 1-10** |
| *bio01* | annual mean temperature (× 10), °C | 3 | – |
| *bio02* | mean diurnal range (mean of monthly (max temp - min temp), °C | 8 | – |
| *bio03* | isothermality [(bio2/bio7) × 100] | – | – |
| *bio04* | temperature seasonality (standard deviation × 100), °C | 6 | – |
| *bio05* | max. temperature of warmest month, °C | 10 | 1 |
| *bio06* | min. temperature of coldest month, °C | – | – |
| *bio07* | temperature annual range (bio5-bio6), °C | 7 | – |
| *bio08* | mean temperature of wettest quarter, °C | 3 | – |
| *bio09* | mean temperature of driest quarter, °C | 10 | – |
| *bio10* | mean temperature of warmest quarter, °C | 11 | – |
| *bio11* | mean temperature of coldest quarter, °C | – | – |
| *bio12* | annual precipitation, mm | 7 | 1 |
| *bio13* | precipitation of wettest month, mm | 9 | 1 |
| *bio14* | precipitation of driest month, mm | – | – |
| *bio15* | precipitation seasonality (coefficient of variation) | 12 | – |
| *bio16* | precipitation of wettest quarter, mm | 9 | – |
| *bio17* | precipitation of driest quarter, mm | 5 | – |
| *bio18* | precipitation of warmest quarter, mm | 4 | 3 |
| *bio19* | precipitation of coldest quarter, mm | 10 | – |
| *bio20* | altitude, m above sea level | – | – |
| *PC1* | first axis of principal component analysis (see Figures S1 and S2) | 11 | – |
| *PC2* | second axis of principal component analysis (see Figures S1 and S2) | – | – |
| *PC3* | third axis of principal component analysis (see Figures S1 and S2) | 4 | 1 |
