# Supplementary material for: Are there morphological and life‐history traits under climate‐dependent differential selection in S Tunesian Diplotaxis harra (Forssk.) Boiss. (Brassicaceae) populations?
Source: Ecol Evol. 2017 Dec 15;8(2):1047–62. doi: 10.1002/ece3.3705 (PMC5773308; doi:10.1002/ece3.3705)
Supplement: Supplementary file 11 [file ECE3-8-1047-s011.doc]

**Table S4.** Environmental variables *bio01* to *bio 20* (see Table S3 for descriptions) for the 12 populations of *Diplotaxis harra* surveyed in the present study, along with results (factor loadings) of principal component analyses (PCA) based on the complete (pop01-pop12) and the reduced (pop01-pop10) dataset, respectively. See also Figures S1 and S2 for the ordination of populations in the two-dimensional space of the PCAs.

| **variable** | **population** | | | | | | | | | | | | **PCA loadings** | | | | | |
| --- | --- | --- | --- | --- | --- | --- | --- | --- | --- | --- | --- | --- | --- | --- | --- | --- | --- | --- |
|  | **1** | **2** | **3** | **4** | **5** | **6** | **7** | **8** | **9** | **10** | **11** | **12** | **populations 1-12** | | | **populations 1-10** | | |
|  |  |  |  |  |  |  |  |  |  |  |  |  | ***PC 1*** | ***PC 2*** | ***PC 3*** | ***PC 1*** | ***PC 2*** | ***PC 3*** |
| *latitude* | 33.770722 | 33.454583 | 33.414972 | 33.485722 | 33.533444 | 33.023917 | 32.922917 | 33.143111 | 33.653722 | 33.886694 | 34.053528 | 34.335639 | 0.11429208 | -0.063263312 | **0.5503374565** | -0.1875427 | 0.14572335 | **0.441012389** |
| *longitude* | 10.815139 | 10.595694 | 10.265472 | 10.060056 | 9.822500 | 10.608056 | 10.397556 | 10.291583 | 10.276194 | 9.716956 | 8.236250 | 8.336750 | **-0.25397421** | -0.008622606 | -0.2469925641 | -0.1603264 | -0.15304328 | **-0.497106031** |
| *bio01* | 199 | 205 | 200 | 187 | 191 | 203 | 199 | 194 | 199 | 197 | 210 | 203 | 0.12364572 | **-0.358353784** | -0.0004201722 | -0.1120513 | **-0.32005316** | -0.038107249 |
| *bio02* | 99 | 117 | 115 | 116 | 118 | 119 | 121 | 119 | 109 | 116 | 129 | 131 | **0.27343551** | 0.041830837 | -0.0950182242 | **0.2889661** | -0.08406655 | 0.003346545 |
| *bio03* | 38 | 39 | 38 | 37 | 37 | 38 | 38 | 37 | 39 | 38 | 37 | 37 | -0.15979850 | **-0.269497839** | -0.0390213065 | -0.1707344 | **-0.23322153** | 0.118019669 |
| *bio04* | 5555 | 6216 | 6357 | 6837 | 6934 | 6550 | 6793 | 6783 | 5892 | 6439 | 7417 | 7426 | **0.26241401** | 0.152507746 | -0.0612707780 | **0.2989846** | 0.07185775 | -0.045647801 |
| *bio05* | 335 | 364 | 356 | 353 | 357 | 367 | 367 | 362 | 343 | 353 | 394 | 391 | **0.27396416** | -0.045797839 | -0.0523881496 | **0.2487481** | -0.16264813 | -0.158560748 |
| *bio06* | 79 | 70 | 60 | 40 | 41 | 60 | 53 | 48 | 67 | 53 | 52 | 45 | -0.16977673 | **-0.316920842** | 0.0615013219 | **-0.2638210** | -0.17448977 | -0.080393606 |
| *bio07* | 256 | 294 | 296 | 313 | 316 | 307 | 314 | 314 | 276 | 300 | 342 | 346 | **0.26955498** | 0.120557212 | -0.0654717011 | **0.3034362** | 0.02619828 | -0.032529168 |
| *bio08* | 175 | 174 | 170 | 109 | 112 | 127 | 120 | 116 | 173 | 165 | 163 | 213 | 0.05280970 | -0.259458036 | **0.4381149771** | -0.2433082 | -0.13538658 | **0.272060020** |
| *bio09* | 266 | 280 | 277 | 271 | 276 | 282 | 281 | 276 | 270 | 275 | 306 | 299 | **0.26950622** | -0.103646857 | 0.0190662562 | 0.2107043 | **-0.22954130** | -0.082695765 |
| *bio10* | 271 | 283 | 280 | 273 | 278 | 285 | 284 | 279 | 275 | 279 | 306 | 299 | **0.26464820** | -0.132921182 | 0.0121598096 | 0.1761482 | **-0.27486421** | -0.052826427 |
| *bio11* | 129 | 124 | 117 | 97 | 100 | 117 | 109 | 105 | 123 | 112 | 116 | 108 | -0.10706959 | **-0.368976076** | 0.0994991151 | **-0.2516622** | -0.19941789 | -0.006480140 |
| *bio12* | 213 | 171 | 163 | 220 | 186 | 139 | 133 | 164 | 175 | 151 | 101 | 135 | -0.22677463 | 0.196949828 | 0.2056033716 | -0.1458718 | **0.29409344** | -0.116329028 |
| *bio13* | 42 | 34 | 26 | 38 | 31 | 23 | 25 | 29 | 33 | 22 | 14 | 16 | **-0.25824030** | 0.107525108 | 0.0784090286 | -0.1958017 | 0.21019079 | **-0.279966817** |
| *bio14* | 0 | 0 | 0 | 1 | 1 | 0 | 0 | 0 | 0 | 0 | 0 | 1 | 0.07839316 | **0.316519593** | 0.2838987480 | 0.1079180 | **0.29197160** | -0.036341173 |
| *bio15* | 74 | 67 | 61 | 60 | 59 | 64 | 63 | 62 | 65 | 56 | 47 | 38 | **-0.26404552** | -0.050254476 | -0.2015795187 | -0.2390576 | -0.06179281 | **-0.407234307** |
| *bio16* | 107 | 79 | 64 | 93 | 77 | 58 | 60 | 69 | 78 | 59 | 36 | 44 | **-0.25718779** | 0.113372586 | 0.1276934996 | -0.2053657 | 0.22491275 | -0.234833014 |
| *bio17* | 2 | 2 | 4 | 8 | 7 | 3 | 3 | 5 | 3 | 4 | 6 | 12 | 0.19060326 | 0.228643753 | 0.2871563622 | 0.1732823 | **0.28269337** | 0.059357585 |
| *bio18* | 19 | 15 | 14 | 17 | 16 | 11 | 9 | 13 | 16 | 16 | 6 | 12 | -0.22463377 | 0.116095088 | **0.3134406468** | -0.1999566 | 0.22912453 | 0.196881234 |
| *bio19* | 74 | 56 | 54 | 79 | 67 | 50 | 49 | 57 | 59 | 53 | 30 | 38 | -0.23643463 | 0.216453601 | 0.0871021361 | -0.1066815 | **0.31706906** | -0.145511434 |
| *bio20* | 24 | 35 | 169 | 455 | 340 | 142 | 293 | 371 | 41 | 70 | 75 | 209 | 0.04123037 | **0.377363262** | -0.1854684112 | 0.2259186 | 0.19771801 | -0.216534744 |
| Proportion of variance explained (%) in PCA | | | | | | | | | | | | | **55.7** | **26.9** | **11.5** | **48.1** | **36.7** | **9.6** |
